# Differential response of the left and right ventricles to pressure overload revealed with diffusion tensor MRI tractography of the heart in vivo

**DOI:** 10.1186/1532-429X-17-S1-O3

**Published:** 2015-02-03

**Authors:** Choukri Mekkaoui, Iris Y Chen, Howard H Chen, William J Kostis, Fabricio Pereira, Marcel P Jackowski, David E Sosnovik

**Affiliations:** 1 Harvard Medical School - Massachussets General Hospital, Charlestown, MA, USA; 2 CHU Nimes, Nimes, France; 3 University of São Paulo, São Paulo, Brazil

## Background

The left ventricle (LV) can tolerate pressure overload from hypertension or aortic stenosis for many years. In contrast, the right ventricle (RV) adapts to pulmonary hypertension only if this develops at birth, while in the adult heart severe and often fatal RV failure develops. We hypothesized that *i*) these divergent responses could be due to differences in myofiber architecture in the free walls of the LV and RV and that *ii*) adaptive changes to pressure overload would be seen in the LV but not the RV.

## Methods

Mice subjected to aortic banding (AB), pulmonary artery banding (PB), and healthy controls, (n=6 per group), were imaged *in vivo*, ensuring that myofiber architecture was imaged under true loading conditions. Diffusion Tensor MRI (DTI) tractography was performed at 9.4T with a 1500 mT/m gradient, a motion-compensated Stejskal-Tanner sequence, 24 gradient directions, b-value of 500 s/mm^2^, and an isotropic resolution of 156 μm^3^[1]. Fiber tracts were color-coded by their helix angle (HA) [2].

## Results

**Figure 1:**
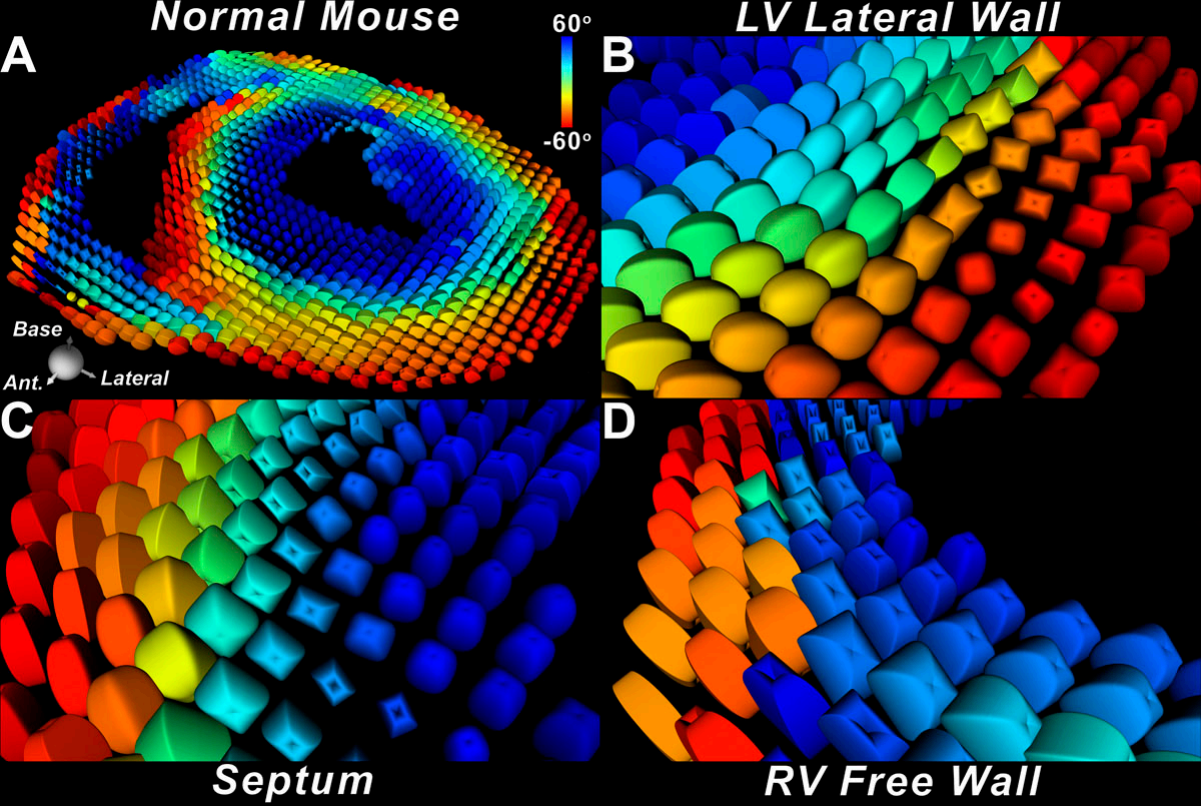
(A) Supertoroidal glyph field of the short axis of a normal mouse heart color-coded by the helix angle (HA). The LV myocardium has a large number of circumferential myofibers, some of which extend a short distance into the RV. However, the bulk of the RV free wall has few circumferential myofibers. (B-D) Magnified view of fiber architecture in the lateral wall (B) and septum (C) of the LV, and in the RV free wall (D). Supertoroidal glyphs are color-coded by HA and show the paucity of circumferential myofibers in the RV free wall and, in contrast, their abundance in both the septum and lateral wall of the LV.

**Figure 2:**
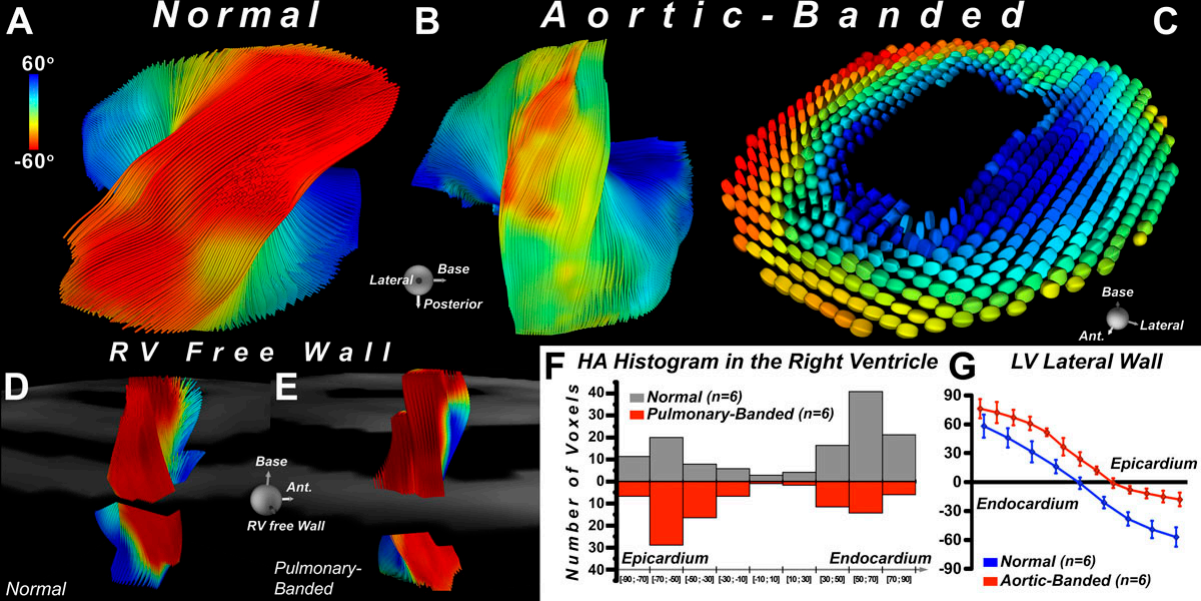
(A) Myofiber tracts in the lateral LV wall of a normal mouse. (B) Rightward shift of myofiber tracts in an aortic-banded mouse. The subepicardial fibers have assumed a more circumferential (yellow *versus* red) orientation. (C) Supertoroidal glyphs demonstrating the rightward shift of myofibers in the LV of an aortic-banded mouse. (D) Transmural evolution of HA in the lateral LV wall of normal and aortic-banded mice. A marked rightward shift is seen in the aortic-banded mice. Tracts in the RV free wall of (E) normal and (F) pulmonary-banded mice appear very similar with few circumferential fibers in either. (G) HA histograms of fibers in the RV free wall of the normal (blue) and pulmonary-banded (red) mice, revealing minimal changes in myofiber organization.

In healthy mice, myofiber organization in the LV was comprised of circumferential myofibers in the midmyocardium, myofibers with a positive HA in the subendocardium, and myofibers with a negative HA in the subepicardium. In contrast, very few circumferential myofibers were present in the RV free wall, which approximated a bilayer. This is illustrated in Figure 1, where fiber architecture in a normal mouse heart is depicted using the supertoroidal model of the diffusion tensor. Marked hypertrophy of the LV/RV myocardium was seen in all banded mice. The LV in all AB mice was hyperdynamic, while in the PB mice the RV was dilated with septal flattening. Fiber architecture in the lateral LV wall of the AB hearts showed a marked rightward shift, transforming myofibers in the subepicardium into a more circumferential orientation (Figure 2A-D). In contrast, the fibers in the RV free wall of the PB mice remained oriented in a near-bilayer with limited change in orientation and few circumferential myofibers (Figure 2E-H).

## Conclusions

The LV contains a large number of circumferential myofibers at baseline and undergoes a rightward shift in fiber architecture in response to pressure overload. In contrast, the RV contains few circumferential myofibers, and its architecture remains largely unchanged in response to increased load. These differences may account for the inability of the RV to tolerate acquired pulmonary hypertension in adulthood.

## Funding

N/A.
